# Magnetic Shielding Accelerates the Proliferation of Human Neuroblastoma Cell by Promoting G1-Phase Progression

**DOI:** 10.1371/journal.pone.0054775

**Published:** 2013-01-23

**Authors:** Wei-chuan Mo, Zi-jian Zhang, Ying Liu, Perry F. Bartlett, Rong-qiao He

**Affiliations:** 1 State Key Laboratory of Brain and Cognitive Science, Institute of Biophysics, Chinese Academy of Sciences, Beijing, China; 2 University of Chinese Academy of Sciences, Beijing, China; 3 Queensland Brain Institute, University of Queensland, Brisbane, Queensland, Australia; University of Saarland Medical School, Germany

## Abstract

Organisms have been exposed to the geomagnetic field (GMF) throughout evolutionary history. Exposure to the hypomagnetic field (HMF) by deep magnetic shielding has recently been suggested to have a negative effect on the structure and function of the central nervous system, particularly during early development. Although changes in cell growth and differentiation have been observed in the HMF, the effects of the HMF on cell cycle progression still remain unclear. Here we show that continuous HMF exposure significantly increases the proliferation of human neuroblastoma (SH-SY5Y) cells. The acceleration of proliferation results from a forward shift of the cell cycle in G1-phase. The G2/M-phase progression is not affected in the HMF. Our data is the first to demonstrate that the HMF can stimulate the proliferation of SH-SY5Y cells by promoting cell cycle progression in the G1-phase. This provides a novel way to study the mechanism of cells in response to changes of environmental magnetic field including the GMF.

## Introduction

All living organisms experience the action of the geomagnetic field (GMF, ∼ 50 µT). Migrating animals and magnetotactic bacteria can make use of the GMF to facilitate their long distance migration and locomotion [1], [2]. A number of experiments have made it obvious that removal of the GMF, i.e. hypomagnetic field (HMF), greatly disturbs the functional state of organisms [3]–[6]. Investigations involving the shielding of biological objects from the GMF provide not only the direct evidence for the biological role of the GMF, but also useful information for the counteractive strategy of the hypomagnetic environments. The environmental magnetic field of outer space is much lower than the GMF and meets the HMF condition: ∼6.6 nT in interplanetary space [7], <300 nT on the moon surface [8], and 0–700 nT 200 km above the ground on Mars [9]. Given the reported adverse impacts of the HMF on many aspects of the living organisms, especially the functions of the central nervous system (CNS), astronauts are exposed to the HMF and thus to potential health risks during interplanetary navigation. An interest in developing ways to counteract the effects of the HMF has consequently arisen, primarily through the study of bio-hypomagnetic responses at the molecular and cellular levels.

HMF exposure has been shown to lead to alteration of the vocal behavior of bird [10] and circadian activity rhythm of bird [11] and rat [12], dysfunction in the learning and memory of *Drosophila* and chicks [13]–[15], a reduction in stress-induced analgesia in mice [16]–[18], and disruption to human cognitive processes [19]. It has been shown that noradrenaline (NA) level in the brain stem of the golden hamster is decreased after HMF exposure [20], and that the effect of the HMF on the CNS is related to a decrease in dendritic spinal density in chicks and a decrease in the density of NA-immunopositive neurons in golden hamsters [20], [21]. Intracerebral injection of exogenous NA can restore the long-term memory of chicks exposed to HMF to a normal level [15]. Investigations with human subjects showed that a 10–day stay in the HMF condition (<50 nT) causes a decrease in visual performance (peripheral critical flicker frequency test) and that shielding of the GMF could also reduce the period of the circadian rhythm [22], [23]. However, standard biochemical and biophysical techniques do not easily allow for an extensive investigation of the broad spectrum of cellular and molecular events. Thus, HMF-triggered neuronal responses at the cellular level remain poorly investigated.

A few studies have reported the effect of the HMF at the cellular level. Studies on cancer cells and plants have found that the HMF can affect both the rate and duration of the cell cycle [4], [24] and that the effects of HMF on human lymphocytes are more significant in G1-phase than G0-phase [25]. In 2000, Sandodze showed that hypomagnetic medium could influence the proliferative activity of the hippocampal fascia dentata and Ammon’s horn suprafimbrial cells in early and late ontogenesis [26]. We have reported previously that cell cleavage during early *Xenopus* development is disturbed and *in vitro* assembly of tubulin is disordered when exposed to the HMF [27], [28]. These results suggest that cell proliferation would possibly be affected by HMF exposure. Nevertheless, a comprehensive analysis of the progression of the cell cycle in the HMF has not yet been reported.

To evaluate the effect of the HMF on the growth of neuronal cells, we designed and constructed a geomagnetic shielding system for cell culture (magnetic fields intensity <200 nT). The proliferation of human neuroblastoma cells (SH-SY5Y), a cell line used commonly in previous neurological studies [29], [30], was examined in this system. We found that the HMF accelerated cell proliferation by promoting the G1-phase progression. This work demonstrates that human neuroblastoma cells can respond to HMF exposure and that G1-phase progression plays a key role during the bio-hypomagnetic interaction process. This method also provides a novel way by which to study the mechanisms underlying the effects of HMF on cell proliferation.

## Materials and Methods

### The Magnetic Shielding System for Cell Culture

**Figure 1:**
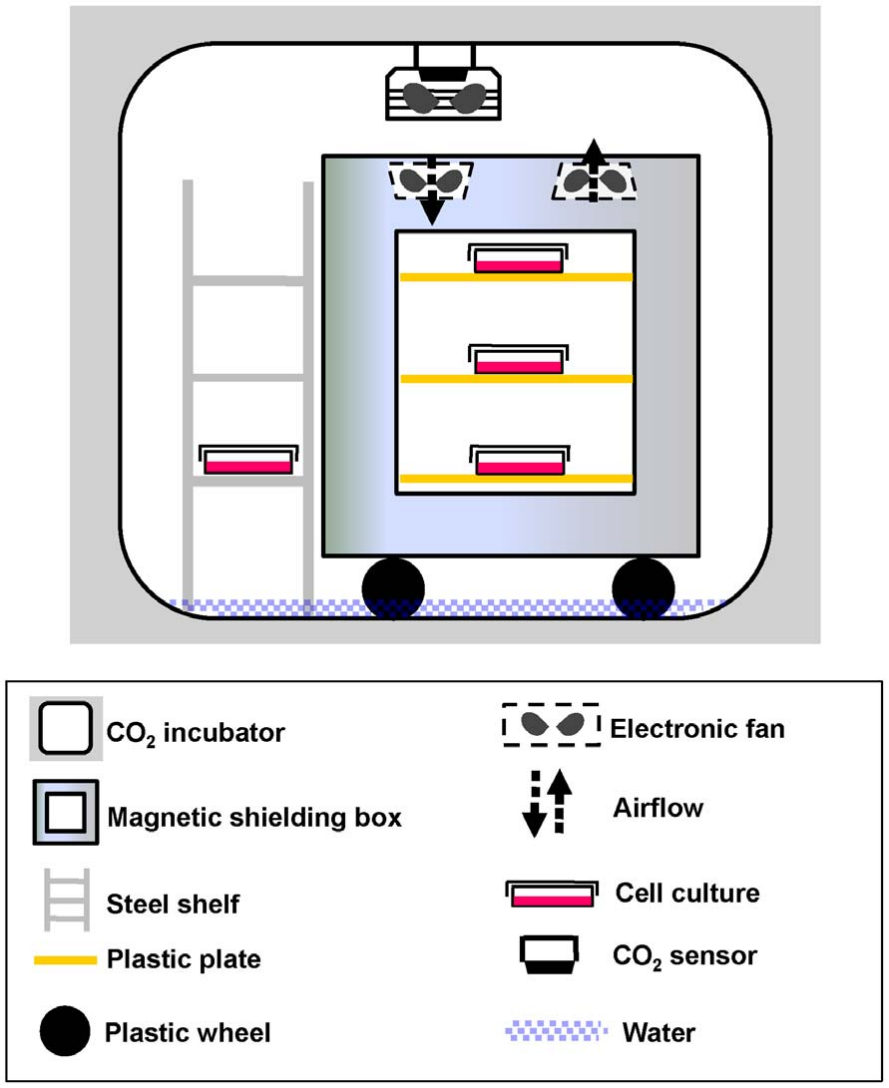
The geomagnetic shielding system for cell culture. A magnetic shielding box was contained in a cell culture incubator. GMF control cells were incubated on the bottom layer of a steel shelf beside the magnetic shielding box. Two fans were installed on the top of the box to facilitate the exchange of gas and temperature between the chambers of the magnetic shielded box and the cell culture incubator.

A permalloy magnetic shielding box was designed for the maintenance of a hypomagnetic condition, as described previously [31]. The dimension of the magnetic shielding box is 470 × 410 × 511 mm^3^ (F-B×W×H). It was constructed with twelve layers of permalloy sheets 0.5 mm thick (magnetic permeability = 20,000, Beijing shougang Company, Beijing, China), enclosed within an outer aluminum layer. The dimension of its inner chamber is 303 × 272×375 mm^3^ (F-B×W×H), which is divided into three layers using plastic plates 100 mm apart. The magnetic shielding box was loaded into a HERA240 cell culture incubator (Thermo Fisher Scientific, Waltham, MA, USA) and a steel shelf was placed beside the magnetic shielding box for the incubation of the non-magnetic-shielded control cells (GMF control). Two fans were installed to ensure that the conditions (gas, humidity and temperature) in the cell culture incubator were identical between the inner and outer spaces of the magnetic shielding box (Figure 1, Table 1). Temperature and relative humidity were measured with a hygro-thermometer (Smart Sensor AR827, Smart Sensor, Hong Kong, China). The CO_2_ concentration was measured with a CO_2_ sensor (Labotec Incubator Control 1050, Labotec, Rosdorf, Germany).

**Table 1:** Cell incubation conditions^a^.

	Temperature(°C)	CO_2_ concentration (%)	Relative humidity (%)
HMF	36.9±0.1	5.1±0.1	97.0±2.0
GMF	37.0±0.1	5.0±0.1	97.8±3.0
GMF’^b^	37.0±0.1	5.0±0.1	97.1±1.0

a Data are mean±s.d. of three measurements at different times;

b The reference geomagnetic field in another cell incubator.

The decay efficiency of the magnetic shielding box was ∼40 dB for the DC magnetic field. The residue magnetic field inside the magnetic shielding box was relatively uniform. The HMF-exposed cells were cultured within the shielding box where the residue magnetic field was lower than 200 nT (Figure S1). The magnetic field of the control shelf varied at different heights and was lower than the GMF in the laboratory (∼36.4 µT) due to the presence of the magnetic shielding box and the magnetic shielding effect of the cell incubator. The average local magnetic field at the bottom control shelf (15.1±2.2 µT) was the highest of the three layers and, therefore, the control cells were placed on this shelf, as indicated in Figure 1. In addition, the magnetic field (56.6±4.4 µT) in another cell incubator (Thermo Forma 371, Thermo Fisher Scientific, Waltham, MA, USA) was taken as a reference field (GMF’ control) in the cell proliferation assay. The direct current (DC) magnetic fields were measured by an APS Model 520 3-Axis Fluxgate Magnetometer (Applied Physics Systems, Mountain View, CA, USA) (Table 2).

**Table 2:** DC magnetic field conditions^a^.

		|B|^b^	|B_x_| c	|B_y_| d	|B_z_| e
HMF (µT)^f^	Top	0.118±0.037	0.057±0.042	0.089±0.030	0.036±0.016
	Middle	0.120±0.044	0.069±0.048	0.087±0.040	0.010±0.008
	Bottom	0.101±0.042	0.049±0.030	0.051±0.035	0.058±0.038
GMF (µT)	Top	10.2±1.1	7.4±3.4	3.4±2.7	4.3±1.5
	Middle	10.8±1.9	9.9±1.3	2.8±2.8	1.4±1.6
	Bottom	15.1±2.2	6.8±2.3	4.2±4.2	11.8±2.5
GMF’ g (µT)		56.6±4.4	50.0±4.6	5.6±5.1	25.4±2.5

a Data are mean±s.d. of measurement reads at the same layer;

b Net DC magnetic field (the vector sum of the three directions);

c Positive direction of the X-axis is pointing from South to North;

d Positive direction of the Y-axis is pointing from West to East;

e Positive direction of the Z-axis is pointing vertically downward;

f Data are from the measurement reads from the cell culture area (|B|<200 nT);

g The reference geomagnetic field in another cell incubator.

Considering the alternative current (AC) magnetic fields generated by the cell incubator and the fans of the magnetic shielding box, we also measured the ambient AC fields in the incubation system with a CCG-1000 induction alternative magnetometer (National Institute of Metrology, Beijing, China) (Table 3). The predominant AC field frequency was checked by a Textronics TDS 2014 digital real-time oscilloscope (Tequipment.NET, Long Branch, NJ, USA). The decay efficiency of the magnetic shielding box was ∼34 dB for the AC magnetic field. The AC fields were 575.7±29.1 nT on the GMF control shelf and 1013.2±157.5 nT in the GMF’ control incubator. The AC field in the magnetic shielding chamber was 12.0±0.0 nT, which was at the same level of the ambient AC field in the laboratory. The predominant frequency was 50 Hz, equal to the power line frequency.

**Table 3:** AC magnetic field conditions^a^.

	|B|^b^(nT)	Dominant Frequency (Hz)
HMF Magnetic shieldingbox	12.0±0.0	50
GMF control shelf	575.7±29.1	50
GMF’ cell incubator	1013.2±157.5	50
Laboratory^c^	14.0±0.0	50

a Data are mean±s.d. of three measurements at different times;

b Net AC magnetic field (the vector sum of the three directions);

c Environmental magnetic field of the room with the incubator.

### Cell Culture

Human neuroblastoma cells (SH-SY5Y cell line; China Cell Resource Confederation, Beijing, China) were maintained in DMEM (High D-glucose) (Gibco/Invitrogen, Grand Island, NY, USA) supplemented with 10% (v/v) fetal bovine serum (FBS; PAA Laboratories, Pasching, Austria), 100 unit/ml penicillin and 100 µg/ml streptomycin (Gibco/Invitrogen, Grand Island, NY, USA) as monolayer in petri dishes (NEST Biotechnology, Wuxi, Jiangsu, China) and the medium was replaced every two days. Cells were detached at sub-confluence with trypsin-EDTA solution (0.25% Trypsin, 0.025% EDTA; Sigma-Aldrich, St. Louis, MO, USA) and re-seeded for subsequent steps. Cells were not used after the 20th passage, as suggested by the supplier. Cells were counted using a hematocytometer (Qiujing Medical Instrument, Yuhuan, Zhejiang, China). Cells were photographed with the Olympus inverted microscope IX71 (Olympus, Shinjuku-ku, Tokyo, Japan).

### Cell Proliferation Assay

Cell proliferation was measured by CCK-8 kit (Dojindo Molecular Technologies, Mashikimachi, Kamimashiki Gun Kumamoto, Japan) and crystal violet staining (Beyotime, Jiangsu, China). For the CCK-8 assay, 200 µl of SH-SY5Y cells (at different densities) were seeded in a 96-well plate, the medium was replaced with 200 µl of fresh medium before the addition of 10 µl CCK-8 solution. The plates were incubated at 37°C for 4 h and the absorbance was read at 450 nm (reference to 630 nm) using a microplate reader (Bio-Rad Laboratories, Hercules, CA, USA).

For crystal violet staining, cells in 6-well plates were fixed with 4% paraformaldehyde (PFA) after incubation at room temperature (RT) for 10 min. After washing the cells twice in phosphate buffered saline (PBS), 1 ml crystal violet staining solution was added to each well and cells were incubated in the solution for 10 min at RT. After washing the cells twice with PBS, the plate was dried completely at RT. 1 ml 2% SDS solution was added to dissolve the cell-binding crystal violet in each well. The crystal violet solution was transferred to a 96-well plate and the absorbance was read at 550 nm using a microplate reader (Bio-Rad Laboratories, Hercules, CA, USA).

### Cell Division Assay

Carboxyfluorescein Diacetate Succinimidyl Ester (CFSE, Sigma-Aldrich, St. Louis, MO, USA) was used to monitor the division of SH-SY5Y cells [32]. Cells were washed twice with DMEM (without FBS), before being incubated in DMEM containing 25 µM CFSE at 37°C for 15 min, at a density of 10^7^ cells/ml. After incubation, the CFSE fluorescence was measured on a Becton Dickinson FACSCalibur flowcytometer with Cell Quest Pro software (BD Bioscience, Franklin Lakes, NJ, USA).

### Flow Cytometry

For cell cycle analysis, cells (2.0×10^4^ cells/cm^2^) were seeded into 60 mm petri dishes. Cells were harvested at certain time points (from 8 h to 52 h with a 4 h interval), washed with ice-cold PBS, fixed in 75% ice-cold ethanol, and re-suspended in 1 ml PBS containing 50 µg/ml propidium iodide (PI; Sigma-Aldrich, St. Louis, MO, USA) and 1 mg/ml RNase A (Sigma-Aldrich, St. Louis, MO, USA). The DNA content was monitored by the flow cytometer, as described above. Cell cycle was analyzed with ModFit LT software (Verity Software House, Topsham, ME, USA).

### Cell Cycle Synchronization

SH-SY5Y cells were synchronized at the G1-phase by serum starvation: 50∼60% confluent cells were transferred into starvation medium (DMEM with 1% FBS) for 72 h incubation. The starved, G1-arrested cells were harvested by trypsinization and transferred into the release medium (DMEM with 20% FBS).

SH-SY5Y cells were synchronized at G1/S border by thymidine double block. 2.5 mM thymidine was added to subconfluent cells seeded in 6-well plates for 20 h. Cells were then washed in PBS 3 times before being released in fresh DMEM (10% FBS) for 9 h. A 4 h secondary thymidine block (2.5 mM) was then performed, after which the cells were again washed with PBS 3 times and placed in fresh DMEM (10% FBS).

SH-SY5Y cells were synchronized at M-phase by nocodazol treatment, which arrests cells at the G2/M-phase by disrupting microtubule assembly. Sub-confluent cells were incubated in DMEM (10% FBS) containing 50 ng/ml nocodazol for 12 h. The cells were agitated and the floating round-shaped cells were collected and re-suspended in fresh DMEM (10% FBS). Cells were seeded at 2.0×10^4^ cells/well in 6-well plates.

### Statistical Methods

Each experiment was repeated at least three times with triplicate samples each time. Means are expressed as mean ± standard deviation (SD). One-way ANOVA was applied for mean comparison. Differences were considered to be significant when p<0.05.

## Results

### GMF Shielding Accelerates the Proliferation of SH-SY5Y Cells

**Figure 2:**
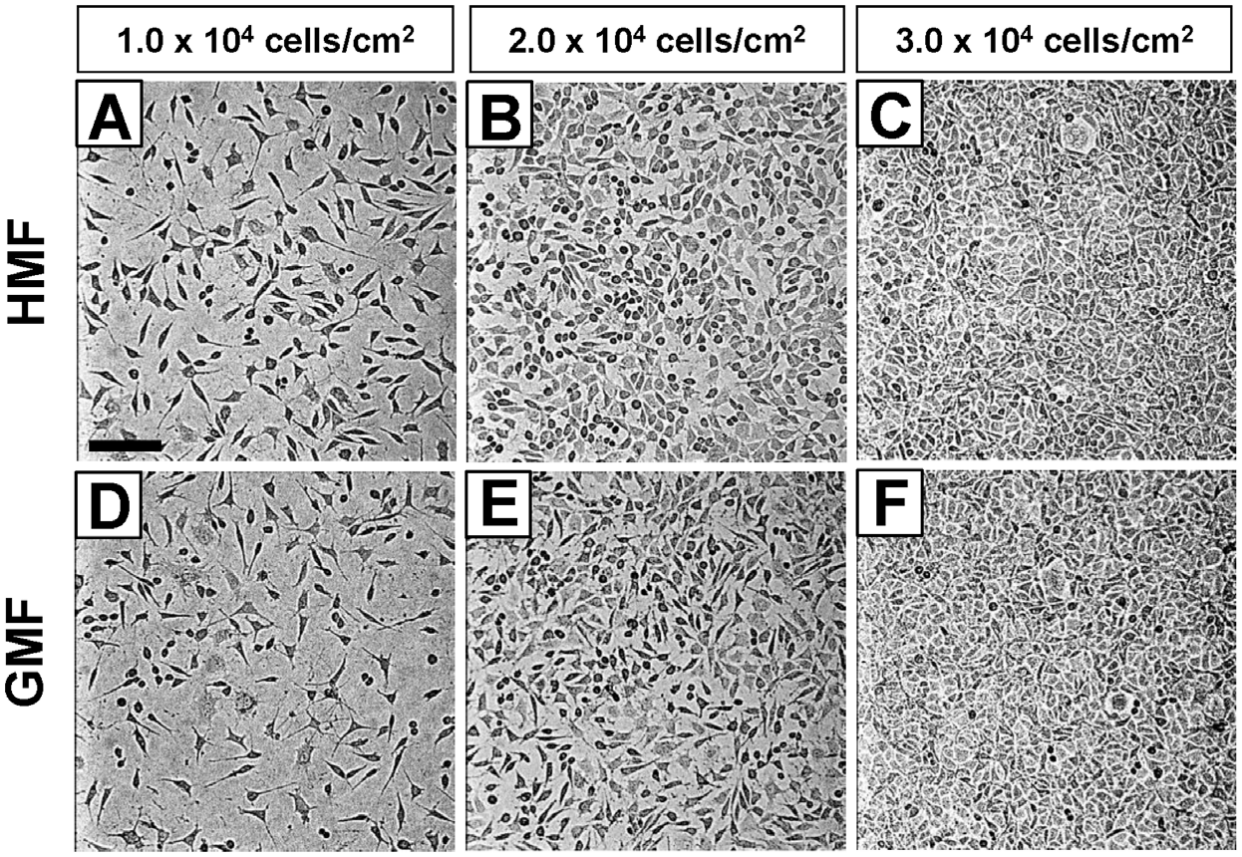
The morphology and density of cells in the HMF. Bright-field images of SH-SY5Y cells seeded at densities of 1.0×10^4^/cm^2^, 2.0×10^4^/cm^2^, and 3.0×10^4^/cm^2^ in 96-well plate after 48 h incubation in the HMF and GMF. Bar = 100 µm.

**Figure 3:**
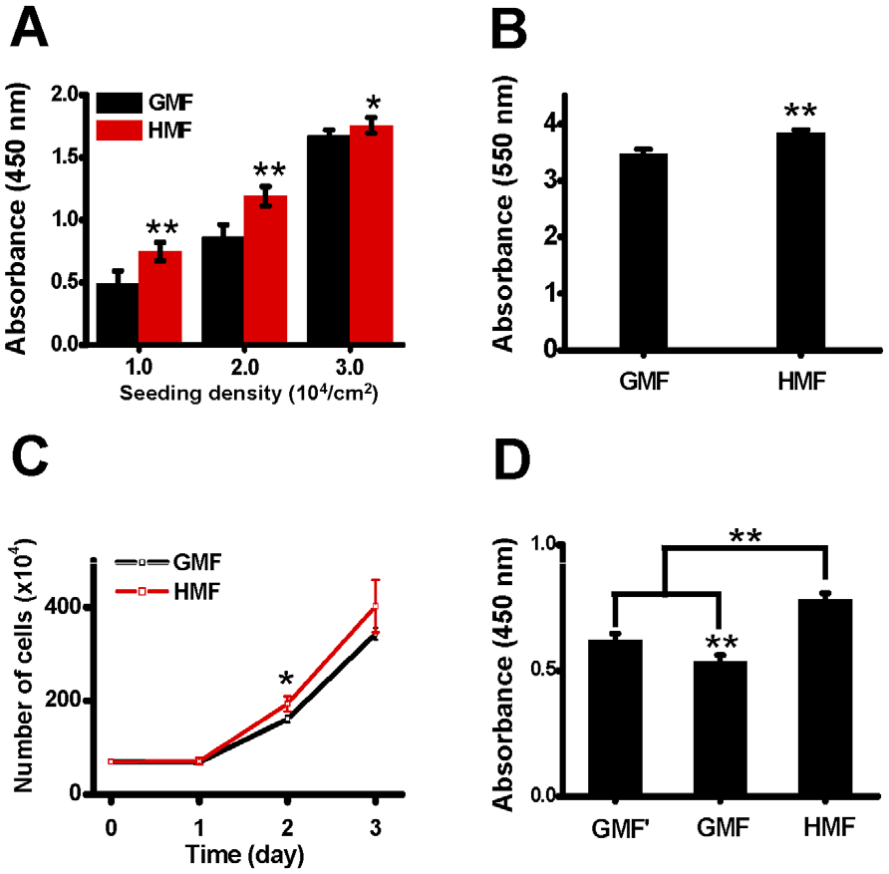
The proliferation of SH-SY5Y cells was accelerated in the HMF. **A:** Cell proliferation assay by CCK-8 kit corresponding to the treatments in (Figure 2) (n = 6). **B:** Cells were seeded at 2.0×10^4^/cm^2^ in 6-well plates and cell proliferation was measured by crystal violet staining after 48 h incubation in the GMF and HMF (n = 6). **C:** Cells were seeded at 2.0×10^4^/cm^2^ in 60 mm petri dishes and incubated for 48 h in the GMF and HMF. The numbers of SH-SY5Y cells were measured at day 1, day 2, and day 3 by hematocytometery (n = 3). **D:** Cells were seeded at 1.5×10^4^ cells/cm^2^ in 96-well plates. Cell proliferation was measured after 48 h incubation in the reference field (GMF’), in the GMF control shelf (GMF), and in the HMF (n = 6). Error bar = s.d.; n = 3; *p<0.05; **p<0.01.

To evaluate the effect of magnetic shielding (the HMF) on the proliferation of neuronal cells, human neuroblastoma cells (SH-SY5Y cell line) were cultured in 96-well plates at 1.0×10^4^ cells/cm^2^, 2.0×10^4^ cells/cm^2^, and 3.0×10^4^ cells/cm^2^ in the magnetic shielding system. After 48 h incubation, no obvious difference in cell morphology was observed between the HMF-exposed cells and the GMF controls. For the groups with 1.0×10^4^ cells/cm^2^ and 2.0×10^4^ cells/cm^2^ seeding densities, the final cell densities in the HMF were higher than the GMF controls (Figure 2). For the groups with 3.0×10^4^ cells/cm^2^ seeding density, cells became over confluent both in the HMF and GMF. CCK-8 assay showed that more viable cells were detected in the HMF conditions than the GMF control, at all seeding densities (Figure 3A). However, the difference in cell proliferation were more remarkable for the groups with lower seeding densities (1.0×10^4^ cells/cm^2^ and 2.0×10^4^ cells/cm^2^, p<0.01).

Next, we measured the change in cell numbers with crystal violet staining and cell counting to confirm the effect of the HMF exposure on cell proliferation. For crystal violet staining, cells were seeded in 6-well plates at 2.0×10^4^ cells/cm^2^. The results showed that significantly more cells (p = 0.0006) were present in the HMF condition (Figure 3B). For the cell counting experiment, cells were seeded at 2.0×10^4^ cells/cm^2^ in 60 mm petri dishes and incubated in the HMF and GMF for 3 days. Cell numbers did not increase until day 1. The number of cells in the HMF was significantly higher than the GMF control at day 2 (p = 0.04), 1.2 times of the control (Figure 3C). Although the number of cells continued to increase after day 2, cells became over confluent at day 3 and the difference between the HMF and GMF groups was not significant. The results indicated that the 48 h HMF exposure promoted cell growth within the experimental conditions.

As mentioned in the Material and Methods, the local magnetic field in the GMF control shelf (∼15.1 µT) was lower than the GMF in the laboratory (∼36.4 µT). To exclude the possibility that the lowered local magnetic field at the control shelf could also affect cell proliferation, we took the magnetic field in another cell incubator as the reference GMF (GMF’) and compared the proliferation of SH-SY5Y cells with CCK8 assay. We noticed that cell proliferation in the GMF’ was higher (p<0.001) than that in the GMF control shelf. However, compared with both the GMF’ and GMF groups, cell proliferation in the HMF was significantly increased (p<0.0001, Figure 3D). This result confirms that the proliferation of SH-SY5Y cells was accelerated under the HMF condition.

### Cell Division was Enhanced in the HMF

**Figure 4:**
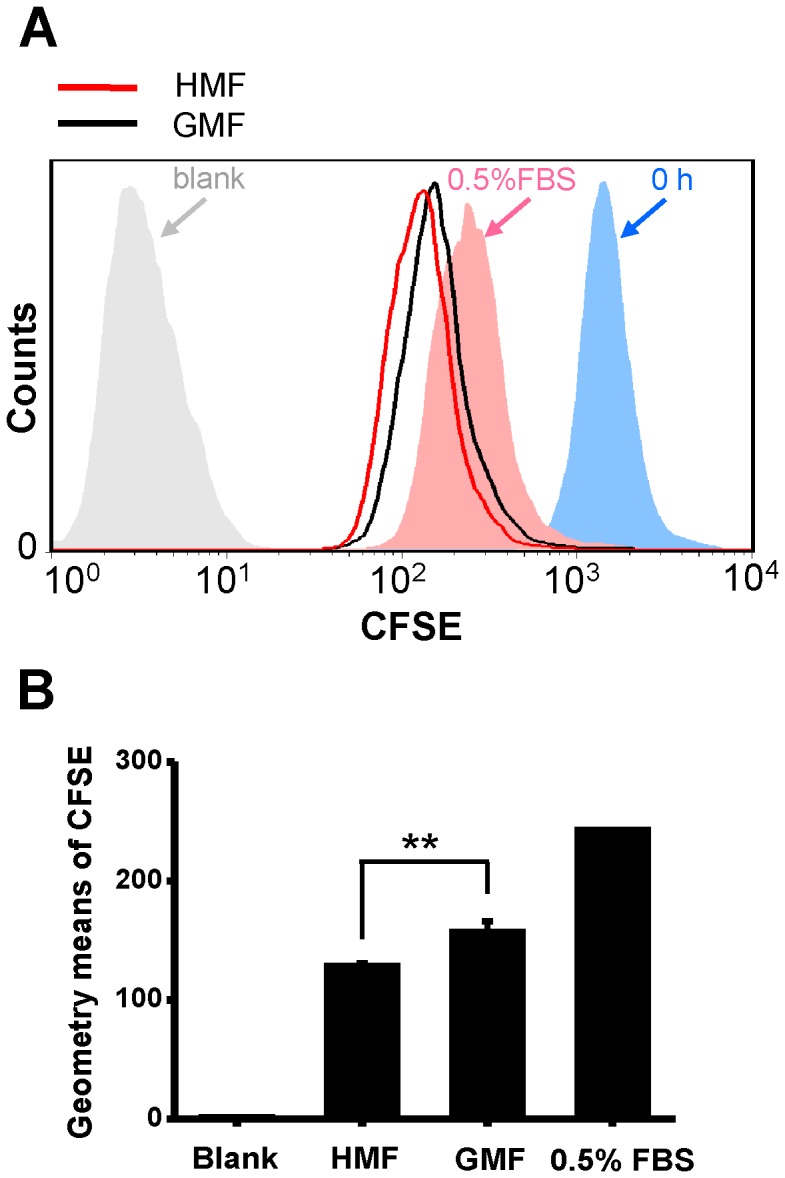
Cell division increases after 48 h HMF exposure. SH-SY5Y cells were stained with 25 µM CFSE and incubated for 48 h in the HMF and GMF. **A:** The fluorescence intensities measured by flow cytometry. CFSE-unlabeled cells were the blank control (grey). CFSE-labeled cells collected immediately after staining (0 h) were the positive control (blue). CFSE-labeled cells incubated in DMEM with 0.5% FBS were the low-proliferation control (pink). **B:** The geometry means of the CFSE fluorescence. Error bar = s.d.; n = 3; **p<0.01.

We monitored the division of SH-SY5Y cells in the HMF after 48 h incubation with CFSE staining. The CFSE-labeled cells were seeded at 2.0×10^4^ cells/cm^2^ into 60 mm petri dishes. CFSE-unlabeled cells were the blank control. CFSE-labeled cells collected before seeding (0 h) were the positive control. CFSE-labeled cells incubated in DMEM with 0.5% FBS were the low-proliferation control. The histogram in Figure 4A showed that the number of cells with weak CFSE-fluorescence in the HMF-exposed cells was higher than the GMF controls. The geometry mean of the CFSE-fluorescence of cells in the HMF was 74% of the controls, significantly lower (p = 0.002) than the GMF group (Figure 4B). The result suggests that the number of cell divisions was higher in the HMF than the GMF control, indicating HMF exposure accelerates cell proliferation.

### The Effect of the HMF on Cell Proliferation is Conditional

**Figure 5:**
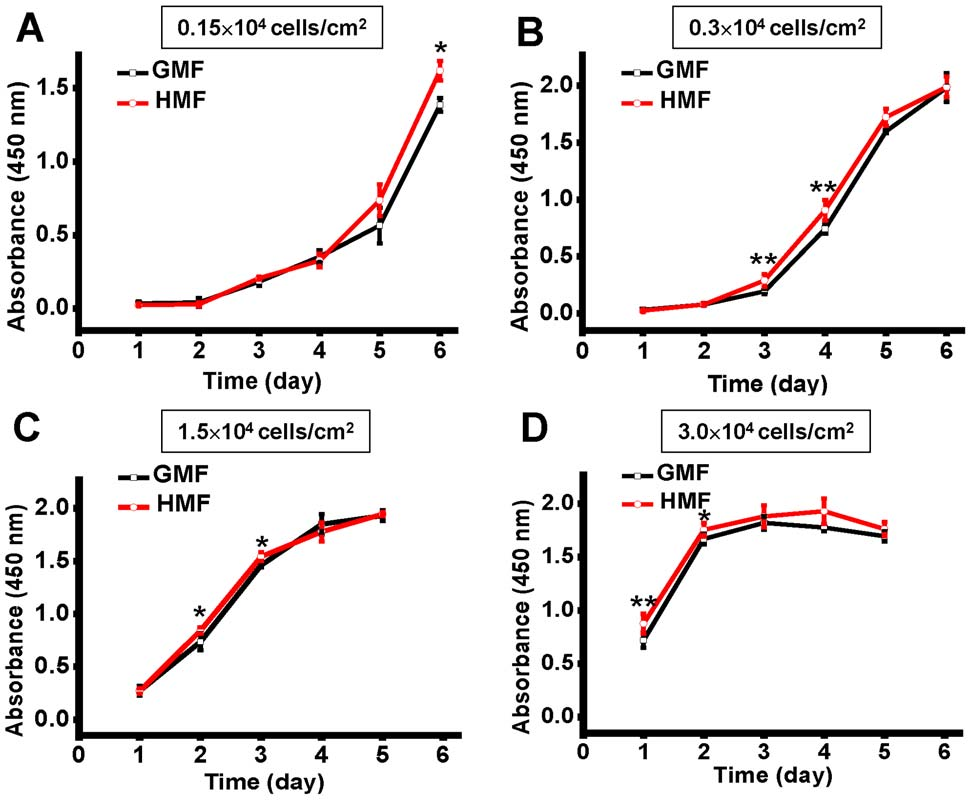
The growth curves of SH-SY5Y cells at different seeding densities. SH-SY5Y cells were seeded in 96-well plates at densities of (A) 0.15×10^4^ cells/cm^2^, (B) 0.3×10^4^ cells/cm^2^, (C) 1.5×10^4^ cells/cm^2^, and (D) 3.0×10^4^ cells/cm^2^. Cell proliferation was measured by CCK-8 assay each day throughout the incubation period. Error bar = s.d.; n = 6; *, p<0.05; **, p<0.01.

The standard incubation condition for the effect of the HMF was evaluated. First, cells were seeded in 96-well plates at a series of densities from 0.15 to 3×10^4^ cells/cm^2^. The dynamic changes in cell proliferation were measured using the CCK-8 assay each day throughout the incubation periods (Figure 5). The growth curves showed that cells in the 0.15×10^4^ cells/cm^2^, 0.3×10^4^ cells/cm^2^, and 1.5×10^4^ cells/cm^2^ groups, reached the logarithmic phase after day 4, day 3, and day 1, respectively. Cells in the 3.0×10^4^ cells/cm^2^ group has already reached the logarithmic phase at day 1. Significant increases in cell proliferation in the HMF were detected under all seeding conditions at the logarithmic phase.

**Figure 6:**
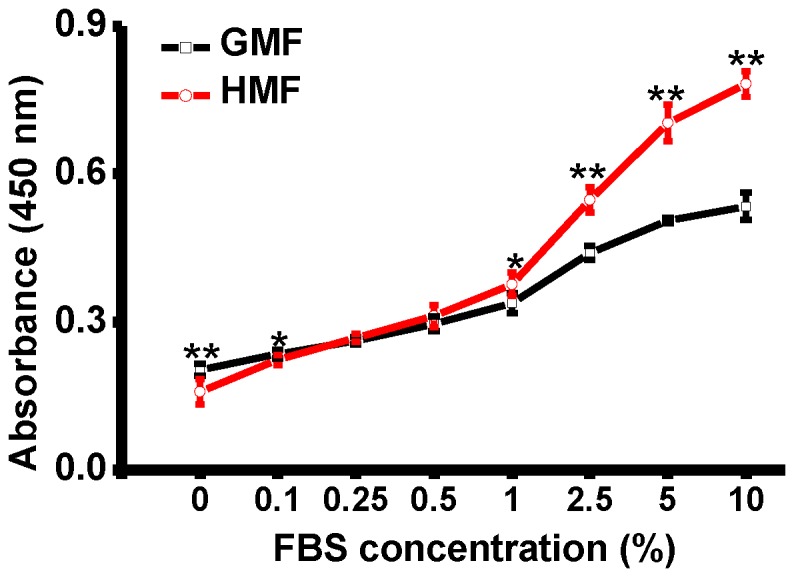
The effect of HMF on cell proliferation depends on the concentration of FBS. SH-SY5Y cells were seeded in 96-well plates at a density of 1.0×10^4^ cells/cm^2^. Cells were incubated in cell culture medium containing different concentrations of FBS (0%, 0.1%, 0.25%, 0.5%, 1%, 2.5%, 5%, and 10%). Cell proliferation was measured using the CCK-8 assay after 48 h incubation in the HMF and GMF. Error bar = s.d.; n = 6; *, p<0.05; **, p<0.01.

Next, cells were seeded at a density of 1.0×10^4^ cells/cm^2^ in 96-well plates in DMEM with a series of FBS concentrations from 0% to 10% (Figure 6). The increase in cell proliferation was detected after 48 h incubation for cells grown in the full culture medium (10% FBS). The CCK assay showed that cell proliferation was decreased when the FBS concentration in DMEM was lowered. However, the stimulative effect of the HMF on cell proliferation was still detectable for cells grown in DMEM with FBS concentrations over 1%. The proliferation of the cells within the HMF and GMF groups were at the same level when the FBS concentration was decreased to 0.25% and 0.5%. Interestingly, the proliferation of HMF-exposed cells was lower than the GMF controls when the FBS concentrations in DMEM were near zero (0% and 0.1%). The results above indicate that the effect of HMF on the proliferation of SH-SY5Y cells also depends on the incubation condition in the culture medium.

The results above show that the effect of the HMF on cell proliferation is conditional. The stages of cell growth and the concentration of FBS in the culture medium will affect the onset of the HMF effect of cell proliferation.

### Cell Cycle Progression is Altered in the HMF

As the HMF affected cell division, we monitored the cell cycle progression of SH-SY5Y cells throughout the incubation period. Cell culture conditions were standardized at 2.5×10^4^ cells/cm^2^ seeding density with DMEM supplemented with 10% FBS in the following experiments.

**Figure 7:**
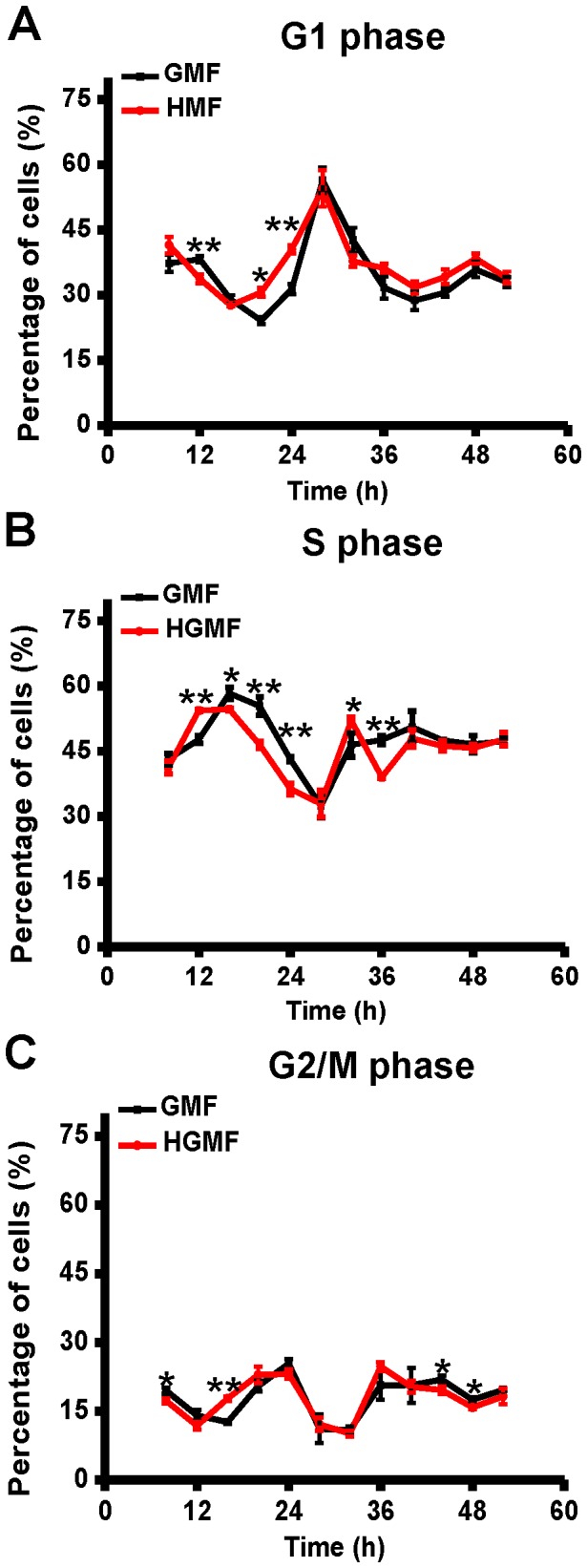
Cell cycle progression of SH-SY5Y cells was altered in the HMF. Cell samples were collected at 4 h intervals from 8 h to 52 h. The DNA content of SH-SY5Y cells was determined by flow cytometry with propidium iodide (PI) staining. The percentage of cells at (**A**) G1-phase, (**B**) S-phase, and (**C**) G2/M-phase was measured. Error bar = s.d.; n = 3; *p<0.05; **p<0.01.

As shown in Figure 7A, the percentage of cells in G1-phase in the GMF started to decrease at 12 h, before increasing at 20 h. The HMF-exposed cells, on the other hand, started to decrease at 8 h and increased at 16 h – a turnaround that was 4 h earlier than the control cells. At 28 h, however, both the GMF and HMF groups reached their maximum, after which both groups maintained a similar percentage of G1-phase cells.

Changes in the percentage of cells in S-phase also showed a 4 h forward shift in the HMF (Figure 7B). The percentage of S-phase cells in the HMF turned from increase to decrease at 12 h, whereas the turning point in the GMF was at 16 h. At 28 h, both groups recorded their minimum number of S-phase cells. Post-28 h a similar level of S-phase cells was maintained, except for a short-term fluctuation from 32 h to 36 h in the HMF.

The percentage of cells in the G2/M-phase started the first transition from decrease to increase at 12 h in the HMF, which was also about 4 h earlier than the GMF controls (Figure 7C). The percentage of cells in G2/M-phase in the HMF was similar to the control from 28 h onwards, as was the case for the G1 and S-phase cells. Although the percentage of cells in G2/M-phase decreased at 44 h and 48 h in the HMF when compared to the control group, the difference in the values was less than 2.0%.

These results show that the cell cycle progression of SH-SY5Y cells was altered in the HMF. A 4 h forward shift of cell cycle progression was observed during the 8–24 h incubation period. This forward shift occurred before the effect of the HMF on cell proliferation could be detected. In addition, we noticed that the response of G1-phase and S-phase cell cycle progression to HMF exposure were more significant than G2/M-phase during the 0–24 h incubation period.

### HMF Promotes G1-phase Progression

G1-phase progression has been reported to be sensitive to the elimination of external magnetic field [24]. Therefore, we investigated the role of G1-phase in the cellular response to the HMF exposure by monitoring the cell cycle progression of G1-phase arrested cells.

**Figure 8:**
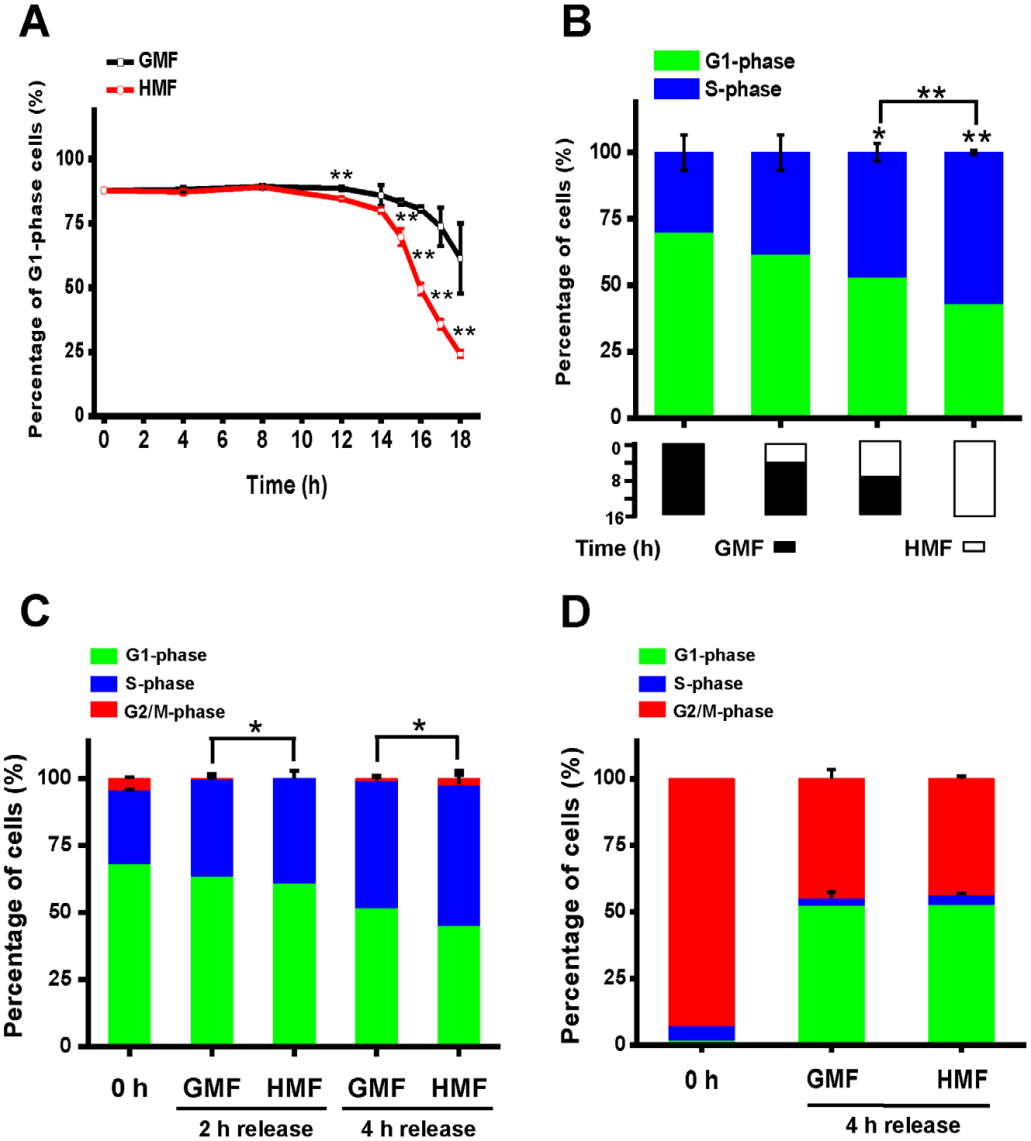
G1-phase progression of SH-SY5Y cells was stimulated in the HMF. A–B: Cells were synchronized at G1-phase by serum starvation before being harvested and seeded in 60 mm petri dishes at a density of 3.5×10^4^ cells/cm^2^. In panel A, the percentage of G1-phase cells was plotted for G1-arrested cells released from 0 h to 18 h. In panel B, G1-phase SH-SY5Y cells were released under four incubation modes: GMF (16 h)**,** HMF (4 h)+GMF (12 h), HMF (8 h)+GMF (8 h), or HMF (16 h). Black blocks indicate incubation periods in the GMF; white blocks indicate incubation periods in the HMF. **C:** Cells were synchronized at the G1/S border phase by thymidine double block. Blocked G1/S border cells were released for 4 h. Cells were harvested at 2 h and 4 h. **D:** Cells were synchronized at M-phase by nocodazol treatment before being seeded and released in either the HMF or GMF in 6-well plates at a density of 1.0×10^4^ cells/cm^2^. Cells were harvested after 4 h incubation. DNA content was determined by flow cytometry with PI staining. Error bar = s.d.; n = 3; *p<0.05; **p<0.01.

Cells were synchronized at G1-phase (∼90%) by 72 h serum starvation. The G1-phase cells were seeded in 60 mm petri dishes at a density of 3.5×10^4^ cells/cm^2^ and incubated in either the HMF or GMF. As shown in Figure 8A, the percentage of G1-phase cells in the HMF and GMF did not change during the 0–8 h period. The percentage of G1-phase cells in the HMF started to decrease at 10 h and was significantly lower than the control cells at 12 h (p = 0.002). It was not until 14 h that an obvious decrease in G1-phase cells was observed both in the HMF and GMF. From 15 h to 18 h, the percentage of G1-phase cells in the HMF decreased significantly compared to the GMF control cells. Although the percentage of G1-phase cells in the GMF also decreased during this period, the rate of decline was much less than in the HMF, indicating that the G1-phase progression was promoted in the HMF. We also compared the progression of G1-phase cells in the GMF, GMF’ and HMF (Figure S2). The data showed that more S-phase and M-phase cells were generated after 24 h release in the HMF.

To evaluate whether the effect of HMF on G1-phase progression depends on the exposure time, G1-phase SH-SY5Y cells were cultured in one of four paradigms: 0, 4, 8, or 16 h HMF incubation followed by a rest period in the GMF. As shown in Figure 8B, with an increase in the HMF exposure time came an increase in the percentage of S-phase cells. Compared to the effect of the 16 h GMF incubation, 8 and 16 h of HMF incubation resulted in a significantly enhanced G1-phase progression (p = 0.02 and p = 0.01, respectively). The effect of 16 h HMF incubation was significantly greater than the 8 h HMF treatment. These results indicate that an 8 h exposure is sufficient to promote G1-phase progression in the HMF, and this effect increases with prolonged exposure time.

In addition, we studied the effect of the HMF on cell cycle progression of late G1-phase cells. Cells were arrested at G1/S border with double thymidine blocking (2.5 mM): 68.3% cells at G1-phase and 27.5% cells at S-phase (0 h) (Figure 8C). After 2 h release, the percentage of G1-phase cells decreased to 60.3±2.8% in the HMF, slightly lower than that in the GMF (p = 0.04; 63.9±1.6%). After 4 h release, the percentage of G1-phase decreased to 42.3±5.6% in the HMF, much lower than 52.5±1.2% in the GMF (p = 0.04). The result showed that HMF was able to stimulate the cell cycle progression of late G1/S border cells within a 4 h exposure period.

The M-phase cell cycle progression in the HMF was also examined. Over 95% of SH-SY5Y cells were arrested at M-phase after 12 h treatment with nocodazol (50 ng/ml; Figure 8D). After 4 h, a small number of S-phase cells were detected, suggesting the newly formed G1-phase cells had started to transfer into S-phase. At this time, around 50% of M-phase cells had entered into G1-phase in both the GMF and HMF groups, and no significant difference could be detected between the M- or G1-phase groups. These results show that the HMF does not alter M-phase progression.

## Discussion

Accumulating evidence has shown that the function of the human CNS, such as circadian rhythm [12], [23], visual sense and cognitive processes [19], [22], could be affected in the HMF. Recent experiments have also revealed that human cells can respond to the magnetic shielding condition. The viability of human spermatozoan cells were increased in the HMF (<500 nT) *in vitro* [33]. HMF exposure can significantly alter cell cycle rates for human cancer-derived cell lines [24]. However, humans are still not believed to have a magnetic sense, especially at the level of GMF. Little evidence is available to demonstrate whether human neuronal cells can respond to the alteration of the environmental GMF [34]. Our results provide direct evidence that the human neuronal cells can in fact respond to the GMF shielding condition. Continuous HMF exposure (48 h) could significantly increase the proliferation of SH-SY5Y cells under standard culture conditions. An obvious forward shift in cell cycle progression of human neuroblastoma cells was observed in the HMF, before the acceleration of proliferation is detectable. We also demonstrate that the effect of the HMF on the proliferation of human neuroblastoma cells is closely related to the progression of the G1-phase and that the pro-proliferative effect of the HMF depends on the exposure time in the HMF, which is the first to present a sophisticated assessment on the relationship between cell cycle progression and HMF exposure. Further investigation of the expression and functional changes of cell cycle related genes in the magnetic shielding system will provide a convenient way to explore the molecular mechanism of the bio-hypomagnetic response.

We observed that the effect of the HMF on cell proliferation differs after different periods of exposure. During 0–24 h, the forward shift of cell cycle progress is significant and easily detected; while, after 28 h, the cell cycle progression was no longer significantly different to controls and could not be distinguished under this experimental condition. Therefore, the timing of observation is important for the analysis of the HMF effect. Our results also showed that the seeding density could affect the effect of the HMF. During the 48 h incubation, groups with low seeding density exhibited more remarkable HMF effect on cell proliferation than the high density group. Martino and colleagues (2010) found that time frame is critical for the observation of the magnetic effect and that the effect of hypomagnetic field on the proliferation of cells seeded at low density is additive for longer incubation time period [24]. Thus, restrict definition of the experimental condition and careful examination on cell cycle progression throughout the incubation period is required for the further investigation of the effects of the HMF.

The effect of the HMF (<1 µT) on the condensation of chromatin in human lymphocytes was observed to be more significant in the beginning of G1-phase [25]. Our results indicate that the acceleration of cell proliferation is led by a forward shift of the cell cycle, and the acceleration of G1-phase progression. We show that in both early and late G1-phase, cells can response to the HMF. As G1/S transition plays an important role in the maintenance of the genomic integrity in mammalian cells [35], we hypothesized that the HMF alters the modification and conformation of the genetic material during the G1-phase. Recently, Martino and colleagues (2011) found that intracellular hydrogen peroxide production in cancer cells and artery endothelial cells was suppressed in the HMF [36]. Reactive oxygen species, especially hydrogen peroxide, has been reported to induce genomic instability in mammalian cells [37], [38]. Thus, further investigation into the changes in genetic material and intracellular reactive oxygen species levels during HMF exposure would be of value.

We have previously shown that the HMF exposure can disrupt the *in vitro* assembly reaction of tubulin [28] and the orientation of the spindle [27]. Nevertheless, this study shows that the cell cycle progression of SH-SY5Y cells arrested at M-phase is not significantly affected in the HMF, suggesting that the *in vivo* dynamics of microtubule during mitosis is not affected. Xiao and colleagues (2009) have shown that the impairment of the learning and memory of chicks in the HMF is related to a decrease in the density of dendritic spines [21]. It is our hypothesis that the structure of microfilament, the basis of cytoskeleton of dendrite spine [39], would be more sensitive to HMF exposure *in vivo*.

Considering adverse effect of the HMF on the functions of the CNS, the acceleration of cell proliferation of human neuroblastoma cells in our HMF system seems not consistent with the previous reports. Many studies have shown that GMF shielding decreases the process of cell proliferation or growth. The growth of exponential phase *Escherichia coli* cells [40] and stationary phase magnetotactic bacterium cells (*Magnetospirillum Magneticum AMB-1*) [41] were decreased in the HMF. The general non-specific response of the root meristems of pea, flax and lentil to hypomagnetic conditions shows an increase in the cell cycle duration [4]. For mammalian cells, long-term HMF exposure can induce atrophic changes in mouse cardiomyocytes [42] and can reduce the proliferation of primary embryonic fibroblasts and increase cell death [43]. Reduction of the GMF to 300 nT leads to the inhibition of proliferation and differentiation of skeletal muscle cells of newborn rat [44]. Martino and colleagues (2010) have shown that HMF exposure (200–500 nT) can significantly decrease the proliferation of human fibrosarcoma (HT1080) and colorectal carcinoma (HCT116) cell lines [24]. However, Borodin and Letiagin (1990) showed that the number of eosinophil granulocytes in C57B1/6 mice increased during 14 days of HMF exposure (<5 nT) [45]. Sandodze (2000) observed both a decrease and an increase in proliferative activity in different cell population of the rat hippocampus after the HMF exposure [26]. We hypothesize that the response of neuronal cells to the hypomagnetic condition depends on the types of cells. Experiments with other neuronal cells, e.g. neurons, glia, microglia, and neural stem cells, especially with primary culture cells, will facilitate an increased understanding of the hypomagnetic effect on the CNS.

Although the local DC magnetic field was successfully shielded in our system, the low frequency ambient AC magnetic field remained. As shown in an earlier study, the low frequency AC noise also could not be completely shielded with high permeability metals (permalloy/μ-metal) [36]. Since the electronic circuits for the maintenance of constant temperature and CO_2_ concentration were inevitably included in the standard design of the cell culture conditions, the AC background in the cell incubator was usually higher than that in the laboratory. However the intensity of AC field in our magnetic shielding box was attenuated to the level of the background field in the laboratory (Table 3). Choleris and colleagues (2002) found that the effect of GMF shielding on stress-induced analgesia in mice obtained in a μ-metal box cannot be reproduced by either compensating the DC component of the GMF with Helmholtz coils or by shielding the AC background with a copper box, suggesting that the AC field plays an important role in the biological effect of HMF [17]. However, our previous experiments showed that the HMFs created by Helmholtz coils and a permalloy shielding room could induce abnormal cleavage in *Xenopus* embryos [27]. Therefore, the effect of ambient AC field and DC field should be discriminated using advanced experimental design in future experiments.

### Conclusions

In summary, human neuroblastoma cells can respond to the HMF depending on the conditions of cell growth. The promotion of cell proliferation is related to an alteration of the cell cycle. The acceleration of the G1-phase plays a particularly important role in the cellular response to the HMF.

## Supporting Information

Figure S1 **The magnetic shielding conditions.** The distribution of the magnetic fields in the magnetic shielding box were plotted according to the vector sum of the magnetic field measurements. The HMF exposed cells were incubated at places with residue magnetic field lower than 200 nT. The white dashed rectangles indicate the areas used for cell culture.
(TIF)

Figure S2 **G1-synchronized SH-SY5Y cells under different magnetic fields.** Cells were synchronized at G1-phase by serum starvation. Cells were released in DMEM with 20% FBS for 24 h at three magnetic fields: GMF’ (∼56 µT), GMF (∼15 µT) on the control shelf, and the HMF. The DNA content was determined by flow cytometry with PI staining. G1-phase cells harvested before releasing were the 0 h control.
(TIF)
