# Supplementary figures and images for: Magnetic Shielding Accelerates the Proliferation of Human Neuroblastoma Cell by Promoting G1-Phase Progression
Source: PLoS One. 2013 Jan 23;8(1):e54775. doi: 10.1371/journal.pone.0054775 (PMC3552807; doi:10.1371/journal.pone.0054775)

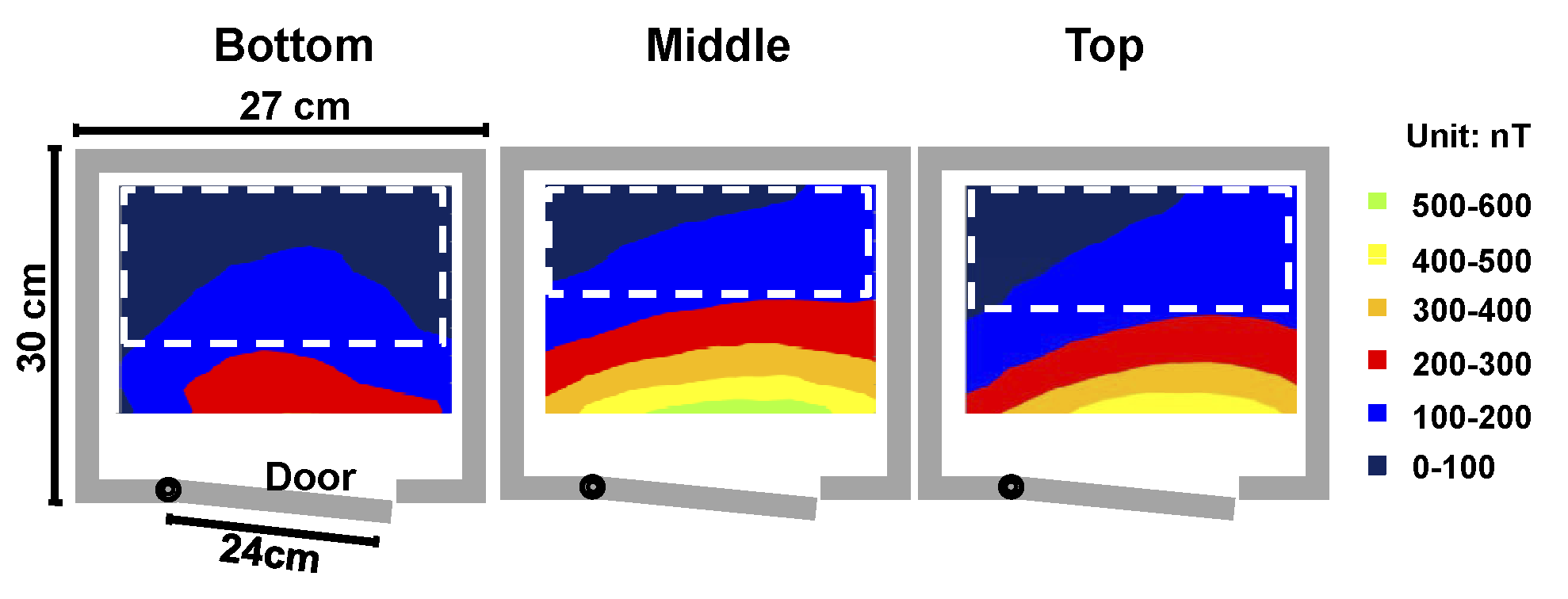

Supplement: Figure S1 — The magnetic shielding conditions. The distribution of the magnetic fields in the magnetic shielding box were plotted according to the vector sum of the magnetic field measurements. The HMF exposed cells were incubated at places with residue magnetic field lower than 200 nT. The white dashed rectangles indicate the areas used for cell culture. (TIF) [file pone.0054775.s001.tif]

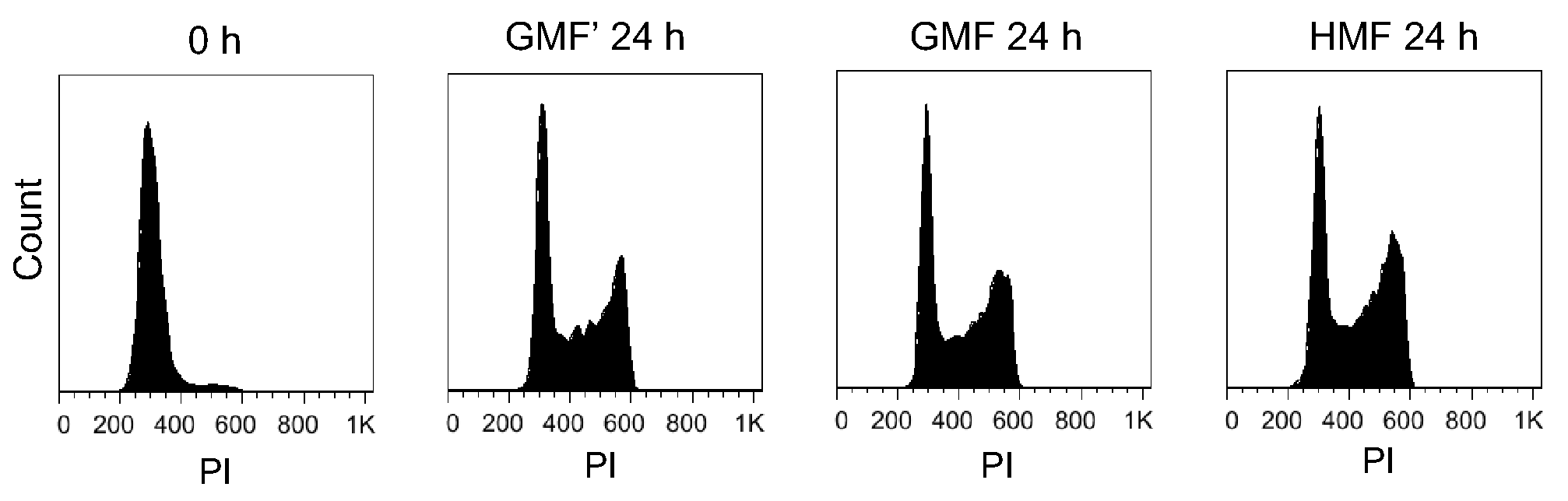

Supplement: Figure S2 — G1-synchronized SH-SY5Y cells under different magnetic fields. Cells were synchronized at G1-phase by serum starvation. Cells were released in DMEM with 20% FBS for 24 h at three magnetic fields: GMF’ (∼56 µT), GMF (∼15 µT) on the control shelf, and the HMF. The DNA content was determined by flow cytometry with PI staining. G1-phase cells harvested before releasing were the 0 h control. (TIF) [file pone.0054775.s002.tif]
